# Do Polish primary care physicians meet the expectations of their patients? An analysis of Polish QUALICOPC data

**DOI:** 10.1186/s12875-020-01190-1

**Published:** 2020-06-23

**Authors:** Anna Krztoń-Królewiecka, Marek Oleszczyk, Adam Windak

**Affiliations:** 1 Department of Family Medicine, Jagiellonian University Medical College, 4 Bochenska Street, 31-061 Krakow, Poland; 2 The College of Family Physicians in Poland, 1 Muranowska Street, 00-209 Warsaw, Poland

**Keywords:** Primary care, general practice, Patient satisfaction, Quality of care, Health services research

## Abstract

**Background:**

Meeting the expectations of patients is one of the most crucial criteria when assessing the quality of a healthcare system. This study aimed to compare the expectations and experiences of patients of primary care in Poland and to identify key patient characteristics affecting these outlooks.

**Methods:**

The study was performed within the framework of the international Quality and Costs of Primary Care in Europe (QUALICOPC) cross-sectional, questionnaire-based study. In Poland, a nationally representative sample of 2218 patients were recruited to take part in the study. As a study tool, we used data from two of four QUALICOPC questionnaires: “Patient Experience” and “Patient Values”.

**Results:**

Patients’ expectations were fulfilled in all study areas: accessibility, continuity, quality of care, and equity. We observed that the highest-met expectations indexes were in the area of quality of care, while the lowest, but still with a positive value, were in the area of accessibility. Patient-doctor communication was the aspect most valued by study participants. Elements of the patient’s own level of engagement during the consultation were ranked as less essential.

**Conclusions:**

Comparing patient experiences to their values allows us to identify areas for improvement that are prioritized by patients. Accessibility is recognized as the most important area by Polish patients, simultaneously showing the highest level of patient-perceived improvement potential. Interpersonal care is another domain, in which the needs of patients are satisfied but are also relatively high. Strong clinician-patient relationships seem to be a priority in patients’ expectations. The continuous efforts in interpersonal communication skills training for primary care physicians should be upgraded.

## Background

Primary health care (PHC) is the part of the healthcare system in which patients address their health concerns first and where the majority of curative and prophylactic healthcare needs of the community are satisfied [1]. PHC responds to the multifaceted health needs of the population, and therefore, it is universal and comprehensive [2]. The organization of PHC differs between countries regarding financing, the scope of services, and the healthcare professionals involved in service provision. In European countries, the model based on the services provided by a family physician or general practitioner is predominant [3]. Gradually increasing expectations of the patients are observed in developed countries [4]. This is accompanied by rapidly increasing costs of healthcare provisions due to the growing burden of chronic diseases and their complications, multimorbidity, and the aging of populations. The World Health Organization emphasizes strengthening the role of PHC as one of the ways to answer these challenges on a global and regional (European) level [5]. Previous research on PHC showed the linkage between PHC accessibility, better coordination, and continuity of care, as well as improved control of healthcare. A possible positive correlation between better access to PHC and better health of the population was also seen [6, 7]. PHC quality is difficult to assess because of its complexity and difficulties in the precise definition of outcomes. The Primary Health Care Activity Monitor in Europe (PHAMEU) study, conducted in 2007–2010, proposed a new approach to determine the quality of PHC. It is based on the classic quality assurance model developed by Donabedian, wherein the framework of structure-process outcome and the features of primary care (PC) (governance, economic conditions, workforce development, access, comprehensiveness, continuity, coordination, quality, and efficiency of primary care) were defined [8].

Patients are assigned three roles in quality assurance: contributors, targets, and reformers [9]. One of the key aspects of the quality of the healthcare system is its ability to meet the needs and expectations of its patients [10]. In Poland, there is a relatively large body of research on patients’ satisfaction with primary care [11–16]. However, existing studies address only single aspects of quality in primary care and do not compare patients’ expectations and experiences. Matching patient’s values with their experiences provide better quality evaluation. If patients have bad experiences with an aspect of care and they value it as very important, this can be assessed as more significant quality problem, which requires improvement, than if the aspect is not considered important [17].

The aim of this study was to compare the expectations and experiences of patients of primary care in Poland. The study was designed to answer the following questions:

1. What are patients’ priorities in primary care, and which aspects are viewed as being less or not at all important?
2. What is the level of patient satisfaction and values of accessibility, continuity, equity, and quality of primary care?
3. Are patients’ personal characteristics related to their expectations?
4. Do patients’ experiences with Polish primary care meet their expectations?

## Methods

### Design of the study

This is a cross-sectional study performed within the framework of the international Quality and Costs of Primary Care in Europe (QUALICOPC) project coordinated by the Netherlands Institute for Health Services Research (NIVEL), aiming to evaluate the quality of PHC in 31 European countries as well as Australia, Canada, and New Zealand [18]. The project took place between March 2010 and February 2014. A set of four questionnaires was developed by the QUALICOPC study team: (1) one for general practitioners (GPs) (“PC Physician” questionnaire), (2) one for patients about their experiences during one specific GP consultation (“Patient Experience” questionnaire), (3) another for patients about the values of PHC they consider important (“Patient Values” questionnaire), and (4) a practice questionnaire about the structure of the PHC setting (“Fieldworker” questionnaire) [19]. The original questionnaires were translated from English to Polish with a formal forward-back translation process. The detailed development of Polish questionnaires, including validation, is available elsewhere [20, 21]. In our study, we used data from two QUALICOPC questionnaires: “Patient Experience” and “Patient Values.”

The study was approved by the Jagiellonian University Bioethics Committee (approval number KBET/104/B/2011).

### Sample

The details of the recruitment of Polish physicians and patients has been described previously [21]. In brief, we approached participating patients through their GPs. A nationally representative sample of 220 primary care physicians was selected from the database of the Polish National Health Fund (the exclusive health insurance company of Poland) by a stratified random sampling procedure. The fieldworker visited the participating PC physician and handed her or him the “GP” questionnaire. Then, in the waiting-room, the fieldworker distributed questionnaires to ten consecutive consenting adult patients visiting the selected physician. The first nine patients completed the “Patient Experience” questionnaire and the tenth one filled out the “Patient Values” questionnaire. The questionnaires were filled out immediately after the appointment with the GP and returned in a sealed envelope to the fieldworker. To realize an aimed response of 2200 patient participants, the fieldworkers had to invite a total of 4663 patients (a response rate of 47%). We excluded two incomplete surveys from data analysis. The final sample included 1979 patients completing the “Patient Experience” questionnaire and 219 patients filling out “Patient Values” questionnaire. To check the representativeness of the participating patients, we compared their age, gender, education level, and employment status to national statistics. Our study population included more women and more patients with secondary and higher-level education than the general population.

### Measures

Items from both questionnaires were assigned to areas of care, according to the key provided by the international study coordinator of QUALICOPC [18]. In this paper, we focus on patients’ experiences and expectations with four primary care areas: “Accessibility” (ACCS), “Continuity” (CONT), “Quality of care” (QUAL), and “Equity” (EQ).

The “Patient Experience” questionnaire asked whether certain performances occurred during the just-completed appointment with the GP. The answering formats of the performance items were “yes” or “no.” In the “Patient Values” questionnaire, patients rated the importance of each of the statements contained in the patient experience survey on a four-point Likert scale from “not important” to “very important.” To compare the questions about experiences with questions about values and expectations, we developed for each studied PC area: a satisfaction index (SI), a value index (VI), and a met expectation index (MEI).

We rescaled all questionnaire items to a uniform scale ranging from − 1 (extremely negative) to + 1 (extremely positive). In the case of dichotomous experience questions (Yes/No), the values (+ 1) or (− 1) were assigned, respectively. For importance questions measured on the Likert scale, values from (− 1) (for “not important”) to (+ 1) (for “very important”) were given; the others were given intermediate values (− 0.5) or (+ 0.5). The satisfaction and value indexes were calculated as arithmetic means (μ) of experience and importance questionnaire items representing particular PC areas. The met expectation index (MEI) for each PC area (A) was computed as a difference between respective satisfaction index (SI) and value index (VI):

$$ {\mathrm{MEI}}_{\mathrm{A}}={\mathrm{SI}}_{\mathrm{A}}-{\mathrm{VI}}_{\mathrm{A}} $$

Additionally, we computed MEI for each experience questionnaire item and a corresponding value questionnaire item. The MEI could range from (− 1) to (+ 1). The values equal to or near 0 indicated complete fulfillment of patient expectations. Negative values pointed to the areas that are very important to patients, but with which they had relatively unsatisfactory experiences.

Socio-demographic data were collected from all patient participants, including gender, age, education level, country of birth, mother’s country of birth, employment status, health status, and presence of chronic disease(s).

### Statistical analysis

Statistical analysis was performed with Statistica 13.1 software (Dell Inc.). To present the results, we used descriptive statistics. To investigate the associations between the values indexes and patients’ characteristics, the Mann-Whitney U test was used for comparing two groups, the Kruskal–Wallis test for more groups, and Spearman’s rank correlation coefficient for two qualitative variables. An α level of *p* = 0.05 was considered for tests of statistical significance.

## Results

### Characteristics of respondents

The mean age of respondents sharing their experiences with PC was 48.2 (SD ± 16.6; min.: 18, max.: 87); women consisted 61%. Nearly half were employed or self-employed; 43% had a middle education level, and 25% had higher education. An average income was declared by 48.5% of respondents, while below-average was declared by nearly 40%. Self-assessed health status was perceived as poor by 17.5% of patients, and just above half had been diagnosed with a chronic medical condition. The mean age of study participants rating the importance was 45.3 (SD ± 14.5; min.: 18, max.: 83 years); women consisted two-thirds of the respondents. Three-fifths of the patients were employed or self-employed, and 56% had a middle education level, while 23% had higher education. An average income was declared by 57% of the study participants and below-average by 30%. Self-assessed health status was declared as poor by 8% of patients, and 41% had been diagnosed with a chronic medical condition.

### Patient expectations

**Fig. 1:**
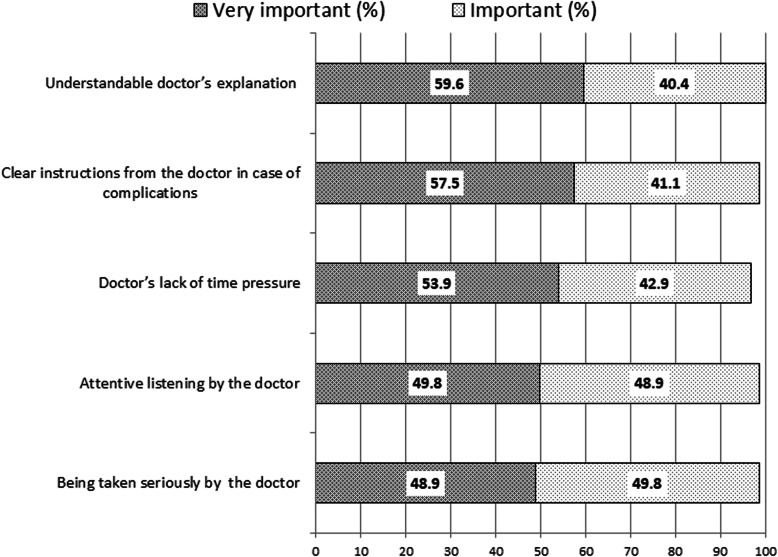
The top five patient expectations

Patient-doctor communication was the aspect most highly valued by study participants. All patients expected that they would clearly understand the doctor’s explanations. The top five expectations of patients **-** proportion of participants who ranked questions as very important and important, are presented in Fig. 1.

**Fig. 2:**
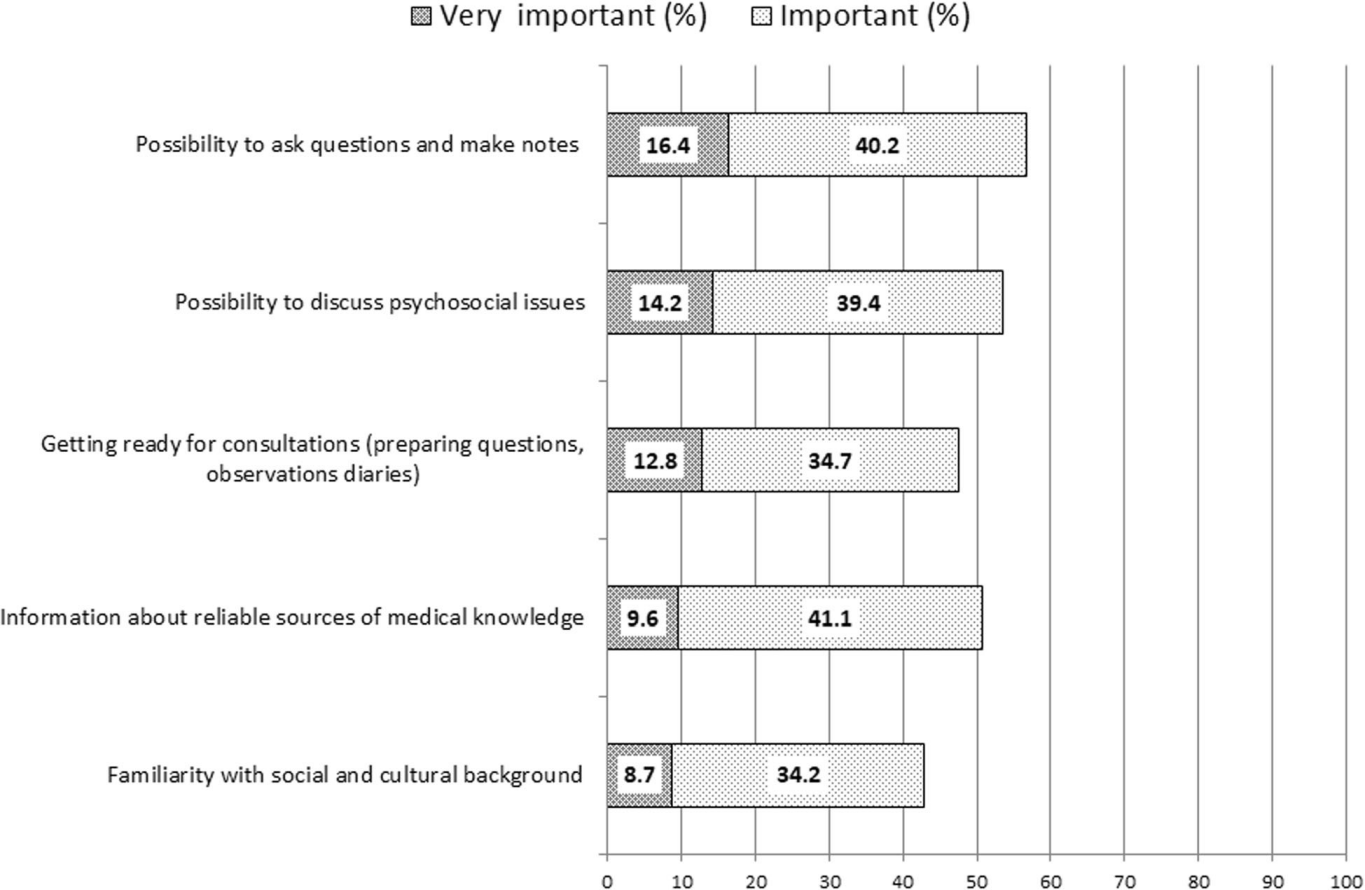
The bottom five patient expectations

The PC physician’s familiarity with patients’ social and cultural backgrounds seemed to be least important for the majority of respondents. Elements of their own engagement in the consultation were also ranked as less essential. Figure 2 shows the bottom five patient expectations **-** proportion of participants who ranked questions as “not important” and “rather not important.”.

### Satisfaction and values indexes

**Fig. 3:**
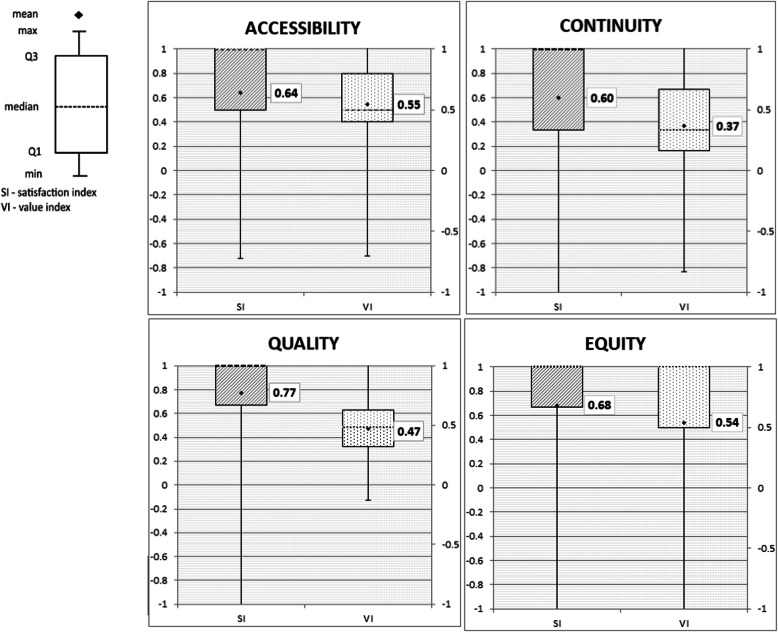
Distributions of the satisfaction and values indexes in particular areas of primary care

In the studied primary care areas, the mean values of the satisfaction index were as follows: “Accessibility,” 0.64 (SD = 0.33); “Continuity,” 0.60 (SD = 0.52); “Quality,” 0.77 (SD = 0,.9); “Equity,” 0.8 (SD = 0.34). The mean values of the value index were as follows: “Accessibility,” 0.55 (SD = 0.35); “Continuity,” 0.37 (SD = 0.39); “Quality,” 0.47 (SD = 0.24); “Equity,” 0.54 (SD = 0.48). The medians and the distribution of the satisfaction and values indexes in particular areas of primary care are presented in Fig. 3.

### Factors affecting values indexes

Accessibility to primary care physicians and continuity of care were significantly more important for patients with chronic conditions than for patients without chronic comorbidities (VI_ACCS_, respectively: 0.60 vs 0.50; *p* < 0.05; VI_CONT_, respectively: 0.47 vs 0.30; *p* < 0.01). The study participants from larger cities had greater expectations concerning accessibility than did patients from rural areas (VI_ACCS_, respectively: 0.63 vs 0.46; p < 0.01). We observed positive correlations between patient age and expectations in 3 of 4 areas: ACCS (r = 0.18; p < 0.01), CONT (r = 0.17; *p* = 0.01), and QUAL (r = 0.22; *p* = 0.001).

### Met expectations indexes

The met expectations index (MEI) values for particular areas were as follows: “Accessibility” 0.096; “Continuity” 0.234; “Quality” 0.299; and “Equity” 0.143.

Table 1 presents SI, VI, and MEI for experience questionnaire items and corresponding value questionnaire items in particular areas of care.

**Table 1:** SI, VI, and MEI for experience questionnaire items and corresponding value questionnaire items

Questionnaire item	Area of care	SI	VI	MEI
**Experiences above expectations**				
Lack of doctor’s prejudices about patient’s gender, religion, or culture	QUAL	0,98	0,31	**0,67**
Doctor’ s politeness	QUAL	0,97	0,66	**0,31**
Doctor’s direct access to patient’s medical record	CONT	0,90	0,63	**0,27**
Doctor’s familiarity with patient’s living conditions	CONT	0,11	−0,15	**0,26**
Eye contact with a doctor	QUAL	0,76	0,51	**0,25**
Extensive opening hours in the practice	ACCS	0,67	0,45	**0,22**
Attentive listening by the doctor	QUAL	0,92	0,73	**0,19**
Politeness and helpfulness of reception desk staff	QUAL	0,84	0,66	**0,18**
Close location of the practice	ACCS	0,69	0,53	**0,16**
Patient’s awareness about out-of-hours medical services	ACCS	0,73	0,57	**0,16**
Doctor’s questions about patient’s health problem(s)	QUAL	0,83	0,67	**0,16**
Doctor’s lack of time pressure	QUAL	0,85	0,73	**0,12**
Doctor’s familiarity with patient’s medical background and health issues	CONT	0,70	0,62	**0,08**
Short waiting time during the phone call to the practice	ACCS	0,59	0,52	**0,07**
Feeling of ability to better handle health problems following doctor’s consultation	QUAL	0,73	0,69	**0,04**
Easy appointment scheduling	ACCS	0,71	0,67	**0,04**
**Experiences equal expectations**				
Understandable doctor explanation	QUAL	0,80	0,80	**0,00**
**Experiences below expectations**				
Doctor’ s interest about other patient’s problems beyond the reason of the visit	QUAL	0,41	0,43	**−0,02**
Engagement of patients in the decisions about their treatment	EQ	0,46	0,54	**−0,08**
Possibility to discuss psychosocial issues with a doctor	QUAL	−0,127	0,039	**−0,166**

*SI* satisfaction index, *VI* value index, *MEI* met expectation index,, *QUAL* quality, *CONT* continuity, *ACCS* accessibility, *EQ* equity

## Discussion

### Summary of main findings

Accessibility, continuity, quality of care, and equity in primary care were important for our respondents – users of PC services, with accessibility being the most crucial area, and continuity the least important. The met expectations indexes for all areas had positive values, indicating the fulfillment of patients’ needs. We observed the highest MEI in the area of quality of care and the lowest, but still with a positive value, in the area of accessibility. Taking into consideration single questions evaluating the particular areas of care, we identified some issues that are important to patients, but with which they have had relatively bad experiences namely: the possibility to discuss with the PC physician not only medical issues but also their personal problems and concerns, their involvement in decision-making, and doctor’s showing an interest in patients’ problems besides the consultation chief complaint.

### Strengths and limitations

This study represents the first exploration of the experiences and values of patients using the services of PC physicians in Poland, based on a representative and a random sample of participants. Our work represents the first time in which such a broad range of data from PHC was collected and within a framework that allows for international comparisons with other countries participating in the QUALICOPC study. The methods used are repeatable, allowing for the monitoring of changes over time if needed. A limitation of the study arose due to the methodology itself, such as the use of questionnaires, which provide indirect data (perceived quality). Possible errors might have occurred at each stage of questionnaire processing. In terms of the design, the chance for error was limited due to cautious and careful multi-stage tool preparation. In data collection, the chance for error was limited due to the use of uniform recruitment strategies and cooperation with professionally-trained fieldworkers. In the analysis stage, the chance for error was limited with the use of automated procedures in data transferring, the employment of a professional statistician, and the use of specialist software. The inclusion criteria limited the findings to the care of adult patients only. The use of stratified sampling instead of general population sampling was another limitation, forced by limited available resources. Differences in the number of participants in both arms of the study (“Experiences” and “Expectations & Values”) might have also influenced the reliability of the findings and our reasoning.

### Findings in light of other studies

The participants of our study most valued the aspect of patient-doctor communication, with understandable explanations from the physician being the most important expectation. These findings are consistent with the results of a systematic review based on an analysis of 19 studies on patients’ priorities for general practice, which found that the most important aspects were: “humaneness” and “informativeness” [22]. Batbaatar et al., in their latest systematic review on determinants of patients’ satisfaction health providers, also confirmed that interpersonal care quality was the essential determinant of patient satisfaction [23]. Patients want professionals who are both interested and sympathetic and provide them with sufficient time and attention [24]. Other QUALICOPC participative countries like Greece and Switzerland also reported the importance of the patient-physician relationship during consultations [25, 26]. The less important patient expectations also showed similarities internationally. Polish patients ranked elements of their commitment to the consultation as less essential. In Switzerland, items related to patients’ activation were generally declared as “very important” by less than 50% of patients [26]. Patient-involvement in decision-making with regards to their treatment plans was scored moderately in Greek data [25]. Patients’ ability to participate in their care may improve medical outcomes [27]. However, multiple studies have addressed the patients’ willingness to be active, and the results are still inconclusive [28–30]. Physicians should assess individual patient preferences to participate in clinical decision making [31]. The experiences of our study participants with being engaged in the decisions about their treatment were reported as being below their expectations.

Among factors affecting patient expectations, we identified age as a major determinant in three of four quality areas. Older patients had higher expectations, but they also were more satisfied with primary care [21]. Participants from larger cities had higher expectations regarding accessibility when compared to patients from rural areas. Contrary findings are presented by Weinhold and Gurtner, who report that there was no significant difference between urban and rural citizens with regards to importance that they placed on accessibility and underline the high importance placed by rural residents on interpersonal relations [32].

### Interpretations of key findings

Patient-centered healthcare delivery is essential for better healthcare outcomes [33, 34]. Skilled communication seems to be the most critical aspect of healthcare provided by primary care physicians [35], as it is the most significant patient expectation and determinant of their satisfaction of care [23]. Strong communication skills deserve equal importance to developing clinical knowledge, procedural skills, and advances in medical technology [36]. Accordingly, the teaching of communication competencies should be fundamental throughout undergraduate and postgraduate medical education [37]. Aspects of the patient-doctor relationship and the physician’s interpersonal communication skills influence patient involvement in decision-making with regards to treatment options [38]. Our study identified a gap between patients’ needs and experiences in the area of patients’ engagement in their care.

## Conclusions

This study evaluates patients’ experiences and values across the four features of primary care: accessibility, continuity, equity and quality of care. Comparing patient experiences to their values allows us to identify areas for improvement that are prioritized by patients. Polish patient needs appear to be fulfilled in all studied areas. Accessibility is recognized as the most important area, simultaneously showing the highest level of patient-perceived improvement potential. However, the possible changes in this area are more dependent on financial and organizational conditions of PHC services, and, therefore, rely on the decisions of health policymakers. Interpersonal care is another domain, in which the needs of patients are satisfied but are also relatively high. Strong doctor-patient relationships seem to be a priority in patients’ expectations of PHC. The continuous efforts in interpersonal communication skills training for PHC physicians should be upgraded. Effective communication directly influences the therapeutic process. Reliable and understandable expressions, in addition to readiness to listen to patient needs are the most valuable communication skills, which should be mastered by every primary care physician in daily practice.

## Data Availability

The data that support the findings of this study are available from NIVEL (The Netherlands Institute for Health Services Research) but restrictions apply to the availability of these data, which were used under license for the current study, and so are not publicly available. Data are however available from the authors upon reasonable request and with permission of NIVEL.

## Abbreviations

ACCS: Accessibility
CONT: Continuity
EQ: Equity
GP(s): General practitioner(s)
MEI: Met expectation index
NIVEL: Netherlands Institute for Health Services Research
QUAL: Quality of care
QUALICOPC: Quality and Costs of Primary Care in Europe
PC: Primary care
PHC: Primary healthcare
PHAMEU: Primary Health Care Activity Monitor for Europe
SI: Satisfaction index
VI: Value index

## References

[1] Starfield B (1994). Is primary care essential?. Lancet..

[2] Starfield B, Shi L, Macinko J (2005). Contribution of primary care to health systems and health. Milbank Q.

[3] Boerma WGW. Profiles of general practice in Europe. Utrecht: NIVEL; 2003. https://www.nivel.nl/sites/default/files/bestanden/profiles-of-general-practice-in-europe.pdf. Accessed 10 July 2019.

[4] World Health Organization (2008). The World Health Report 2008: primary health care now more than ever.

[5] World Health Organization (2011). Developing the new European policy for health - Health 2020.

[6] Shi L, Macinko J, Starfield B, Politzer R, Wulu J, Xu J (2005). Primary care, social inequalities and all-cause, heart disease and cancer mortality in US counties: a comparison between urban and non-urban areas. Public Health.

[7] Friedberg MW, Hussey PS, Schneider EC (2010). Primary care: a critical review of the evidence on quality and costs of health care. Health Aff.

[8] Kringos DS, Boerma WGW, Bourgueil Y, Cartier T, Hasvold T, Hutchinson A (2010). The European primary care monitor: structure, process and outcome indicators. BMC Fam Pract.

[9] The DA, Lecture L (1992). Quality assurance in health care: consumers’ role. Qual Health Care.

[10] Doyle C, Lennox L, Bell D (2013). A systematic review of evidence on the links between patient experience and clinical safety and effectiveness. BMJ Open.

[11] Kurpas D, Steciwko A (2002). Quality of primary health care and patients’ satisfaction. Wiad Lek.

[12] Marcinowicz L, Chlabicz S (2006). Improvement in the accessibility and organization of services of family physicians in a small town in Poland: a comparison of patient opinions between 1998 and 2002. Adv Med Sci.

[13] Marcinowicz L, Konstantynowicz J, Chlabicz S (2008). The patient’s view of the acceptability of the primary care in Poland. Int J Qual Heal Care.

[14] Marcinowicz L, Rybaczuk M, Grebowski R, Chlabicz S (2010). A short questionnaire for measuring the quality of patient visits to family practices. Int J Qual Heal Care..

[15] Miller M, Supranowicz P, Gebska-Kuczerowska A, Car J (2007). Evaluation of patients’ satisfaction level as a part of the quality of primary health care functioning. Pol Merkur Lekarski.

[16] Marcinowicz L, Grębowski R, Chlabicz S (2009). Exploring negative evaluations of health care by polish patients: an attempt at cross-cultural comparison. Health Soc Care Community.

[17] Jung H, Wensing M, de Wilt A, Olesen F, Grol R (2000). Comparison of patients’ preferences and evaluations regarding aspects of general practice care. Fam Pract.

[18] Schäfer WL, Boerma WG, Kringos DS, De Maeseneer J, Gress S, Heinemann S (2011). QUALICOPC, a multi-country study evaluating quality, costs and equity in primary care. BMC Fam Pract.

[19] Schäfer WL, Boerma WGW, Kringos DS, De Ryck E, Greß S, Heinemann S (2013). Measures of quality, costs and equity in primary health care instruments developed to analyse and compare primary care in 35 countries. Qual Prim Care.

[20] Krztoń-Królewiecka A, Oleszczyk M, Schäfer WLA, Boerma WG, Windak A (2016). Quality of primary health care in Poland from the perspective of the physicians providing it. BMC Fam Pract.

[21] Oleszczyk M, Krztoń-Królewiecka A, Schäfer WLA, Boerma WGW, Windak A (2017). Experiences of adult patients using primarycare services in Poland – a cross-sectional study in QUALICOPC study framework. BMC Fam Pract.

[22] Wensing M, Jung HP, Mainz J, Olesen F, Grol R (1998). Which aspects of general practice care are important for patients? A systematic literature review. Soc Sci Med.

[23] Batbaatar E, Dorjdagva J, Luvsannyam A, Savino MM, Amenta P (2017). Determinants of patient satisfaction: a systematic review. Perspect Public Health.

[24] Walsh S, Arnold B, Pickwell-Smith B, Summers B (2016). What kind of doctor would you like me to be?. Clin Teach.

[25] Lionis C, Papadakis S, Tatsi C, Bertsias A, Duijker G, Mekouris PB (2017). Informing primary care reform in Greece: patient expectations and experiences (the QUALICOPC study). BMC Health Serv Res.

[26] Droz M, Senn N, Cohidon C (2019). Communication, continuity and coordination of care are the most important patients’ values for family medicine in a fee-for-services health system. BMC Fam Pract.

[27] Frosch D, Kaplan R (1999). Shared decision-making in clinical medicine: past research and future directions. Am J Prev Med.

[28] Doherty C, Stavropoulou C (2012). Patients’ willingness and ability to participate actively in the reduction of clinical errors: a systematic literature review. Soc Sci Med.

[29] Davis RE, Sevdalis N, Vincent CA (2011). Patient involvement in patient safety: how willing are patients to participate?. BMJ Qual Saf.

[30] Schildmeijer K, Nilsen P, Ericsson C, Broström A, Skagerström J (2018). Determinants of patient participation for safer care: a qualitative study of physicians’ experiences and perceptions. Health Sci Rep.

[31] Levinson W, Kao A, Kuby A, Thisted RA (2005). Not all patients want to participate in decision making. A national study of public preferences. J Gen Intern Med.

[32] Weinhold I, Gurtner S (2018). Rural - urban differences in determinants of patient satisfaction with primary care. Soc Sci Med.

[33] Benson BJ (2014). Domain of competence: interpersonal and communication skills. Acad Pediatr.

[34] Davis K, Schoenbaum SC, Audet AM (2005). A 2020 vision of patient-centered primary care. J Gen Intern Med.

[35] Sari MI, Prabandari YS, Claramita M (2016). Physicians’ professionalism at primary care facilities from patients’ perspective: The importance of doctors’ communication skills. J Family Med Prim Care.

[36] Warnecke E (2014). The art of communication. Aust Fam Physician.

[37] Franco CAGDS, Franco RS, Lopes JMC, Severo M, Ferreira MA (2018). Clinical communication skills and professionalism education are required from the beginning of medical training - a point of view of family physicians. BMC Med Educ.

[38] Smith SK, Dixon A, Trevena L, Nutbeam D, McCaffery KJ (2009). Exploring patient involvement in healthcare decision making across different education and functional health literacy groups. Soc Sci Med.

